# Climate change is affecting mortality of weasels due to camouflage mismatch

**DOI:** 10.1038/s41598-018-26057-5

**Published:** 2018-05-24

**Authors:** Kamal Atmeh, Anna Andruszkiewicz, Karol Zub

**Affiliations:** 1 Université de Bordeaux, 351 Cours de la Libération, 33400 Talence, France; 2 Univ Lyon, Université Lyon 1, CNRS, Laboratoire de Biométrie et Biologie Evolutive UMR5558, F-69622 Villeurbanne, France; 3 Mammal Research Institute of the Polish Academy of Sciences, Stoczek 1, 17-230 Białowieża, Poland

## Abstract

Direct phenological mismatch caused by climate change can occur in mammals that moult seasonally. Two colour morphs of the weasel *Mustela nivalis* (*M*. *n*.) occur sympatrically in Białowieża Forest (NE Poland) and differ in their winter pelage colour: white in *M*. *n*. *nivalis* and brown in *M*. *n*. *vulgaris*. Due to their small body size, weasels are vulnerable to attacks by a range of different predators; thus cryptic coat colour may increase their winter survival. By analysing trapping data, we found that the share of white subspecies in the weasel population inhabiting Białowieża Forest decreases with decreasing numbers of days with snow cover. This led us to hypothesise that selective predation pressure should favour one of the two phenotypes, according to the prevailing weather conditions in winter. A simple field experiment with weasel models (white and brown), exposed against different background colours, revealed that contrasting models faced significantly higher detection by predators. Our observations also confirmed earlier findings that the plasticity of moult in *M*. *n*. *nivalis* is very limited. This means that climate change will strongly influence the mortality of the *nivalis*-type due to prolonged camouflage mismatch, which will directly affect the abundance and geographical distribution of this subspecies.

## Introduction

In the last decade, the number of studies on the adaptation of mammals to changing environmental conditions has increased remarkably due to rapid climate change^1–5^. Continuous warming is altering both seasonal and multiannual^6,7^ natural cycles, which is significantly impacting the distribution and fitness of various species^1,8^. However, there is increasing evidence that there exist sufficient phenotypic traits and underlying genetic variation to enable rapid adaptation to changing climatic conditions^9,10^.

Camouflage mismatch in seasonal coat colour is a major consequence of climate change^5^ and may result in selective predation pressure, which can be an important outcome of natural selection^5,11,12^. Studies on snowshoe hares (*Lepus americanus*) that considered different climate change scenarios have shown that phenotypic mismatch in seasonally moulting species will increase in the future^5^. This would lead to extreme decreases in individual fitness if no evolutionary or adaptational changes occur^13^. Many animals have limited moulting plasticity; thus to respond to changing climatic conditions they exhibit many behavioural adaptations to minimise the fitness costs due to increased predation pressure. For instance, foraging willow ptarmigans *Lagopus* 1. *lagopus* select habitats characterised by lower food quality, but which offer optimal camouflage^14^, whereas rock ptarmigans *Lagopus mutus* change from conspicuous to cryptic by soiling their plumage^15^. However, for other species (e. g. snowshoe hare) there is no evidence of behavioural adaptations for minimizing predation risks relating to colour mismatch^16^. Thus there is a need for experimental work as well as observational/correlative studies to investigate camouflage mismatch in all moulting species to understand the demographic consequences of such stressors.

The main aim of our study was to examine how camouflage mismatch and moult plasticity affect the fitness and distribution of individuals representing different phenotypes in two colour morphs of the least weasel *Mustela nivalis*. In weasels, two main subspecies (distinct colour morphs) have been described, which occur in different climatic conditions^17^. In Poland, the first one − *Mustela nivalis nivalis* occurs only in the east and at higher latitudes in the mountains, while the second − *Mustela nivalis vulgaris* occurs in the west and south. Recently, *M*. *n*. *vulgaris* has expanded to the north and east, and both morphs currently occur sympatrically in several places, e.g. in Białowieża Forest (K. Zub, unpublished data). These morphs also exhibit some variation of the patterns of their summer colouration, but the differences between their winter pelages are far more important, as *M*. *n*. *nivalis* is characterized by a white winter coat, whereas *M*. *n*. *vulgaris* has a brown coat all year round^18^ (Fig. S1).

Due to their small body size, weasels are very vulnerable to attacks from other predators, mainly raptors^19^. In some years, predation can be the largest mortality factor of weasels^20^. Thus, cryptic coat colour may reduce predation pressure and increase survival, especially in autumn/winter and spring^13^. Our main hypothesis is that due to increasing phenotypic mismatch, which might be caused by further climate change, the proportions of both colour morphs of weasel will change according to the prevailing weather conditions. In the extreme scenario, if the *M*. *n*. *nivalis* morph is not capable of responding to climate change by shifting its moulting time, it will either disappear locally or shift its range, and be replaced by *M*. *n*. *vulgaris*. Alternatively, further climate warming will cause a stronger selection pressure on *M*. *n*. *nivalis*, leading to a change in moulting pattern and improved survival probability of this morph under new environmental conditions.

To test this hypothesis, we analysed the effect of winter weather conditions on the survival of different morphs of weasels from trapping data. Next, we performed an experiment to examine the effect of camouflage mismatch on weasel mortality. Finally, we analysed the plasticity of moulting in *M*. *n*. *nivalis* in order to investigate the potential of adaptation of this morph to changing climatic conditions. Based on the results of this study, we propose possible explanations for the present distribution of different weasel morphs and potential shift of their ranges due to climate change.

## Results

**Figure 1:**
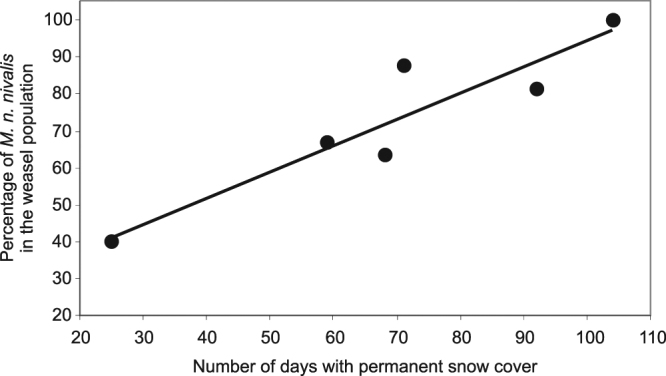
Relationship between the share of *M*. *n*. *nivalis* in summer/autumn population of weasels inhabiting the Białowieża Glade and number of days with permanent snow cover during the preceding winter (data for years 1997, 2003–2007).

From 1997 to 2007 we captured 118 weasels, including 23 of *M*. *n*. *vulgaris*. Analysis of the live-trapping data showed a significant positive relationship between the summer-autumn share of *M*. *n*. *nivalis* in the weasel population inhabiting the study area and the number of days with snow cover during the preceding winter (LM, F_1,4_ = 23.93, R^2^ = 0.82, p < 0.01, Fig. 1).

During the field experiment, we recorded 138 model-predator encounters with a 31.9% rate of detection. The red fox was the principal predator, followed by the raccoon dog. Avian predators were rarest, and were mainly buzzards (Buteo buteo and Buteo lagopus) and ravens (Corvus *corax*) (Table 1).

**Table 1:** Numbers of each predator (red fox *Vulpes vulpes*, raccoon dog *Nyctereutes procyonoides*, grey wolf *Canis lupus*, common raven *Corvus corax*, buzzard *Buteo buteo* and *Buteo lagopus*) recorded by camera-traps at experimental sites and numbers of detected models (camouflaged and non-camouflaged).

	Red fox	Raccoon dog	Grey wolf	Common raven	Buzzard	**Sum**
Predator presence	95	29	6	6	2	**138**
White model detected	26	2	2	2	—	**32**
Camouflaged	—	—	—	—	—	**—**
Non-camouflaged	26	2	2	2	—	**32**
Brown model detected	10	—	—	1	1	**12**
Camouflaged	6	—	—	1	—	**7**
Non-camouflaged	4	—	—	—	1	**5**
**Total number of models detected**	**36**	**2**	**2**	**3**	**1**	**44**

We found that camouflage was the most significant factor affecting detection by a predator: weasel models with incompatible camouflage were detected more often than those matching the background colour (Table 2). Detection rates also decreased with increasing percentage of snow cover, but this effect was not significant (Table 2). Neither duration of experimental session nor model colour significantly affected detection rate (Table 2). Random variables in the model were year and site identity (ID), nested within experimental session, but both were non-significant (likelihood ratio <0.000006, df = 1, p = 0.99 and likelihood ratio = 1.399, df = 3, p = 0.706, respectively). After removing the non-significant fixed terms from the model (see methods section), the effect of snow cover also became significant (GLMMs, z = −2.148, p = 0.032).

**Table 2:** Effect of camouflage (camouflaged or not), area covered with snow (0–100%), duration of experimental session (days) and model colour (brown or white) on weasel model detection.

Predictor	Estimate	SE	z	P
Camouflage (yes)	−2.816	1.021	−2.759	0.006
Percentage snow cover (%)	−0.182	0.111	−1.647	0.099
Duration of experimental session (days)	0.071	0.058	1.236	0.216
Model colour (white)	−0.272	0.975	−0.279	0.780

Results were based on generalized linear mixed models (GLMM), with binomial distribution for the dependent variable.

**Figure 2:**
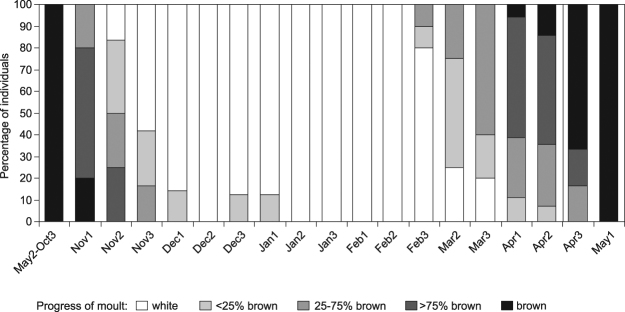
Progress of moult in *Mustela nivalis nivalis* in Białowieża Forest. Each bar indicates the proportion of individuals at different stages of moult, summarized for the years 2002–2017 (N = 130 observations of moulting animals). Each month was divided into 10-day periods. From the beginning of May until the end of October only brown individuals were present in the population. Percentage of brown colour refers only to the dorsal part of the weasel body (see Fig. S2 for details).

The analysis of moult progress revealed that in autumn most animals completed their moult within 40 days (from the beginning of November until the first days of December), whereas the spring moult was much longer (around 90 days, from the end of February until the end of April, Fig. 2).

From our observations, we concluded that the period over which most individuals had a winter coat lasted around 130 days. We compared this data with historic records of weasel skins in the collection of the Mammal Research Institute (years 1965–2017) and did not find any deviation from this pattern, i.e. all animals moulted within the time limits predicted by our observations (Fig. 2). Moreover, from examining this collection we found that *M*. *n*. *vulgaris* was first recorded in Białowieża Forest in 1997, after which it was seen regularly.

**Figure 3:**
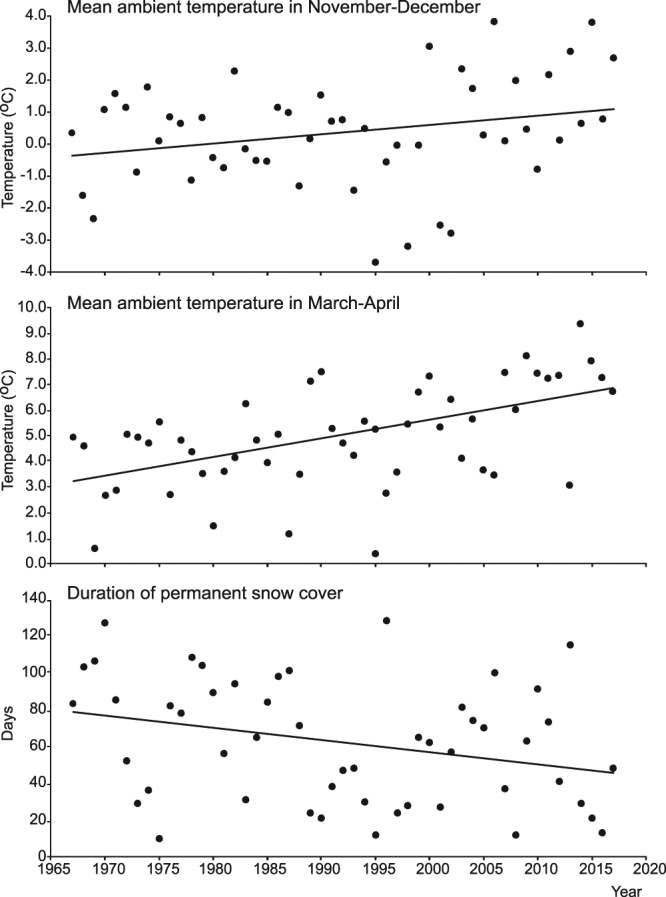
Temporal variation of mean ambient temperatures in November-December (autumn moult of weasels), mean ambient temperatures in March–April (spring moult of weasels) and duration of permanent snow cover during the last 50 years (1967–2017) in the Białowieża Forest.

Analysis of weather data collected in Białowieża Forest over the last 50 years (between 1967 and 2017) revealed that the autumn moult correlates with the beginning of permanent snow cover (snow depth >5 cm lasting for at least 5 days) in this area (on average 20th December, 95% CI: 14th December – 26th December). On the other hand, in spring, permanent snow cover ends on average on the 2nd March (95% CI: 14th February –10th March), thus before all animals complete their moults (Table S1). During the analysed period, the mean number of days with permanent snow cover decreased from around 80 days down to only 40 days (Table S1; LM, F_1,49_ = 4.82, p = 0.03, R^2^ = 0.07, Fig. 3). This was mainly caused by the overall shortening of winter: at the beginning of the studied period snow completely disappeared on average on the 16th March, whereas in recent years it has been disappearing on the 21^st^ February (LM, F_1,49_ = 4.84, p = 0.03, R^2^ = 0.07). Over the same period, there has been no change in the start of permanent snow cover (LM, F_1,49_ = 0.01, p = 0.927, R^2^ = 0.00). A second factor that has contributed to the decrease in the mean number of days with permanent snow cover is the occurrence of mid-winter snow-free breaks, which last on average 31.8 days (SD = 14.33 days, range 15–56 days). During the analysed period, the frequency of mid-winter snow-free periods has increased significantly (LM, F_1,49_ = 4.40, p = 0.041, R^2^ = 0.06), as in the past they happened on average once every 3.4 years, whereas recently they have been occurring once every 1.9 years (Table S1). Mean temperatures in November-December (autumn moult) have not changed significantly over the last 50 years (LM, F_1,49_ = 3.47, p = 0.069, R^2^ = 0.07), whereas during the analogous period, in March-April (spring moult) mean temperature has increased on average by 3.6 °C (LM, F_1,49_ = 23.54, p < 0.001, R^2^ = 0.32, Fig. 3, Table S2).

## Discussion

In the Białowieża Forest weasel population, the proportion of *M*. *n*. *nivalis*, a morph that changes its pelage colour to white in winter, decreased along with a simultaneous decrease in the number of days with snow cover in the region. Furthermore, the results of the field experiment with differently coloured weasel models revealed that detection decreased when the model was camouflaged and when snow cover increased in the study area. These results support the hypothesis that predation pressure can be one of the main natural factors affecting the selective mortality of weasels in winter. However, our predictions are based only on experiments with artificial models, whereas live animals may exhibit many anti-predatory behaviours. Thus the actual selective pressure on individuals with mismatched seasonal camouflage could be much lower, but probably high enough to cause local changes in the abundances and distributions of both weasel morphs. In any case, artificial models have been successfully used for a long time to study the effects of camouflage mismatch on the mortality of different animals^11,12^.

Our results are in accordance with previous studies conducted on camouflage mismatch, confirming the general negative effect of this factor on seasonally moulting species^21,22^. Snowshoe hares display limited phenotypic plasticity in their winter brown-to-white moult, causing increasing camouflage mismatch with decreasing snow cover^5,23^. This led to high fitness costs due to predation in poorly camouflaged individuals^13^. Similarly, increased levels of predation due to camouflage mismatch were found in mountain hares (*Lepus timidus*), where the number of individuals decreased with decreasing snow cover in winter and increasing generalist predator abundance^23^.

Our observations also confirmed earlier findings that the plasticity of moult in *M*. *n*. *nivalis* is very limited^18^. This means that climate change will strongly influence the mortality of the *nivalis*-type due to prolonged camouflage mismatch, which will directly affect the numbers and geographical distribution of this subspecies. However, the limited plasticity of weasels during the autumn moult most probably results from the relatively unchanged ambient temperatures in November-December, which affect the onset of pelage change. The most important factor driving the process of moult in mustelids is a hormonal cascade induced by photoperiod^21^, but ambient temperatures also modify this process^24–26^. Thus as early winter temperatures (November–December) have been relatively stable over the last fifty years, there has been no reliable signal to cause weasels to delay pelage change from brown to white. In contrast, spring mean ambient temperatures have been gradually increasing, which has caused weasels to moult earlier. A future increase in early winter temperatures caused by climate warming could potentially delay moulting in *M*. *n*. *nivalis*, which would shorten the time of camouflage mismatch and decrease their mortality.

The mortality due to predation of both morphs decreases with increasing depth of snow cover, because weasels extensively hunt under the snow^18^. Thus, *M*. *n*. *nivalis* should suffer higher mortality than *M*. *n*. *vulgaris* when there is a lack of snow cover, but difference in survival between the two morphs of weasels will be lower when snow cover is high enough to enable them to hide.

Considering the limited plasticity of moult timing in weasels and further climate warming leading to decreasing snow cover, we suppose that *M*. *n*. *nivalis* could disappear from some areas and be entirely replaced by *M*. *n*. *vulgaris*. Adaptive evolution of moulting time in *M*. *n*. *nivalis* is also further constrained by the fact that the decreasing number of days with snow cover is not only caused by the overall shortening of winter, but also by a substantial increase in the frequency of mid-winter snow-free periods. Therefore, even if weasels are able to modify their response to photoperiod^27^, they are unable to reverse the progress of moulting when weather conditions change during winter.

The first records of *M*. *n*. *vulgaris* in the study area coincided with a relatively long series of mild winters at the beginning of the 1990’s, but this morph never overran *M*. *n*. *nivalis*. Further progress in the expansion of *M*. *n*. *vulgaris* may be limited by physiological constraints. Previous studies have demonstrated that weasels are able to adjust their metabolic rates according to actual ambient temperatures in order to save energy or avoid overheating^28–30^. However, there is very little data about the physiological ability of *M*. *n*. *vulgaris* to adapt to different climatic conditions. Our unpublished data suggests that there are systematic differences in metabolic rate levels between *M*. *n*. *nivalis* and *M*. *n*. *vulgaris*, with the latter usually characterized by a higher resting metabolic rate (RMR). This may restrict the presence of this form in the generally harsher conditions of Central and Eastern Europe. Nonetheless, more data on the physiological mechanisms of both subspecies is required in order to understand the potential shifts of their ranges and population distributions in future climatic conditions. Moreover, camouflage plays a very important role for weasels when they cover long distances on the snow’s surface. Thus when snow is present, *M*. *n*. *vulgaris* probably minimizes its exposure to predation, which may in turn limit its ability to exploit new hunting areas or limit its speed when moving, because of the necessity to stay under the cover of snow. This may lead to higher mortality of this subspecies, as weasels’ energy demands are high^29^.

We demonstrated that camouflage mismatch in seasonal coat colour can result in selective predation and be an important factor in natural selection. According to our results, increased mortality due to camouflage mismatch may lead to local extinction of the white winter morph and replacement by the brown one. If a change in winter pelage colour is an adaptive trait, there should be an association between mortality (fitness measure) and the genes (or alleles) that determine winter whitening. Thus future research on seasonally moulting animals should focus on the genetic component of this process and potential for evolutionary adaptations^9,10,12,22^. More knowledge is also needed to understand what other factors than photoperiod and ambient temperature can modify the timing of moult and affect the plasticity of this trait.

## Methods

### Study area

This study was carried out in the central part of the Białowieża Forest (23.86°E, 52.70°N), north-east Poland. The area encompassed three types of habitats: the first is the pristine forest of the Bialowieza National Park (BNP), which is dominated by oak-lime-hornbeam forest. The second habitat comprised meadows and unmanaged grasslands to the north of Białowieża village and surrounded by forest, arable land and settlements. The river valley stretching along the Narewka River formed the third habitat, with diverse plant communities (see^20^ for details). A variety of predators are present in the Białowieża Forest and many of them regularly hunt weasels^31^. The medium-sized predator community is dominated by red fox (*Vulpes vulpes*), pine marten (*Martes martes*), raccoon dog (*Nyctereutes procyonoides*), common buzzard (*Buteo buteo*), lesser-spotted eagle (*Clanga pomarina*) and tawny owl (*Strix aluco*)^31^.

### Weasel trapping

We live-trapped weasels at three sites (Forest, Meadows and River valley) between 1997 and 2007. Trapping was done once in spring (April–June), three times in summer (July–September), once in autumn (October–November) and twice in winter (December–February). We set 20 to 40 traps, about 50 m apart, on transects (1.0–2.0 km of length) following linear features of the landscape, which are preferred by weasels, such as fences and ditches^31^. Each trapping session lasted 5–7 days and traps were checked twice a day during the whole period. Following the procedure described by^32^, weasels were marked before being released at the place of capture. Based on summer and autumn data (highest numbers of captured animals, see^20^), we estimated the proportions of both morphs in the population.

### Camouflage and predation

To determine how camouflage affects mortality, we recorded predation events on brown and white stuffed models (Gosig mus, IKEA Article Number 501.454.73). Models were modified to make them longer and slimmer with a shorter tail to maximise resemblance to a weasel (Fig. S3). We exposed models against contrasting backgrounds, either on bare ground not covered with snow or on the snow layer. Hence, when weather conditions were stable, we placed camera traps (Bushnell Natureview HD MAX) at different locations, with two models, one of each colour, placed approximately three to five meters apart and about ten meters from the camera (Fig. S3). Cameras were triggered by motion sensing and were programmed to record 15-second videos after each taken picture. We conducted the experiments during the following time intervals: March–April 2015, January–March and November–December 2016. Each recording session lasted from one to two weeks at 4 to 6 sites located at least 100 meters apart, and chosen randomly in the open meadows of the study area. The locations of the experiments were changed randomly after every session. A brown model on snow and a white model on snow-free ground implied camouflage mismatch, whereas the opposite was considered completely cryptic. Detection was confirmed when the following predator behaviours occurred on the models: taking, attacking, urinating, sniffing and observing. To avoid any speculation, events where the models were taken without any camera record, or where the predator’s behaviour was unclear, were removed from the analyses. Predators from the same species were considered as two distinct individuals whenever there was a discontinuity in the time of detection and if no distinguishing characteristic was found.

### Moult progress

To analyse the progress of moulting in *M*. *n*. *nivalis* individuals we used data collected between 2002 and 2017, using 45 video-records and 85 trapping-records. For the video-records, we were unable to distinguish between individual animals, but for the trapping-records we included the same individuals (in total 64 different animals) if they were trapped at different stages of moult. From January 2014 till February 2016, twelve special camera traps were distributed evenly at the three different sites (Białowieża National Park, open grasslands and Narewka river valley). We placed the traps 500 meters apart at fixed locations, following linear features (roads, fences and riverbanks). Cameras (Bushnell Natureview HD MAX) were triggered by motion sensing and were programmed to record 15-second long videos. Camera-traps worked continuously, and the data was retrieved every 2–3 weeks. For every video-recorded individual we recorded the date and time, type of morph (*nivalis* or *vulgaris*) and moulting stage (summer fur, winter fur, moulting). We classified all individuals into five groups: completely white (winter), dorsal part less than 25% brown, dorsal part 25–50% brown, dorsal part >75% brown, or completely brown dorsal part of the body (summer) (Fig. S2). Finally, we estimated the proportion of individuals at different moulting stages (5 groups) during every 10-day period of each month. Data on snow cover duration was collected from the meteorological station in Białowieża village. We used climate data collected between 1967 and 2017, which approximately corresponds with the records of weasel skins in the collection of the Mammal Research Institute PAS in Białowieża.

### Statistical analysis

For live-trapping, we used a Linear Model (LM) to separately study the relation between *M*. *n*. *nivalis* share in the summer-autumn population (dependent variable) with number of days with snow cover during the preceding winter (explanatory variable). Another linear model was performed to study both the progress of snow cover over the years and changes of mean ambient temperatures during the spring and autumn moult, in which the year was used as an explanatory variable. Both linear models were checked for normality of residuals, homoscedasticity and over-dispersion.

We restricted our field experiment’s analyses to the situations when, according to the video-recordings, there was a predator present that either ignored or detected at least one model. To assess the main effect of camouflage on detection, we used a Generalized Linear Mixed Model (GLMM) with a binomial distribution for the response variable (0 = undetected, 1 = detected) and logit link function. The fixed explanatory variables were as follows: camouflage as a two-level factor (0 = not camouflaged, 1 = camouflaged), model colour (two-level factor, brown or white), duration of experimental session (days) and percentage of area covered by snow. We were able to estimate snow cover during each of the experiments from the pictures and videos, not in terms of depth but as a range of values from 0 to 100%. The random variables used in the model were as follows: site number (models were present at 4–6 sites during each session) nested within experimental session and year. The significance of random variables was estimated using the log likelihood ratio test. Both random variables were non-significant, but we decided to keep them in the model to control for the independence of observations (there were two models - brown and white, present at every site). When performing the initial analyses, we also used interactions between the fixed terms, but they turned out to be non-significant; thus we did not include them in the final analyses. All analyses were performed using R Studio 1.0.44.

### Data availability

Climatic data and description of experimental design are given in the Supplementary files. Additional information is available on request.

### Ethical statement

Weasels were captured and handled in strict accordance with the guidelines set by the Polish Committee on the Ethics of Animal Experiments. The protocol was approved by the Local Committee on the Ethics of Animal Experiments at the Medical University in Białystok (permit numbers LKE 04/2003, LKE 06/2004 and LKE 49/2014). Weasels are protected in Poland and were trapped under the auspices of Polish nature conservation authorities (permits no. DOPweg- 4201-04-6/03/jr, DOPog-4201-04-43/05/aj, WPN.6401.155.2014.MW and DLP-III-4102-36B/26349/14/KG).

## Electronic supplementary material

Table S1, Table S2

Supplementary information
