# Supplementary material for: Climate change is affecting mortality of weasels due to camouflage mismatch
Source: Sci Rep. 2018 May 24;8:7648. doi: 10.1038/s41598-018-26057-5 (PMC5967304; doi:10.1038/s41598-018-26057-5)
Supplement: Supplementary file 1 — Table S1, Table S2 [file 41598_2018_26057_MOESM1_ESM.doc]

Table S1. Duration of permanent snow cover (snow depth > 5 cm lasting at least for 5 days) in the Białowieża Forest in years 1967-2017.

| Winter period | Permanent snow cover | | | | Permanent snow cover (days) | Mid-winter snow-free period (days) |
| --- | --- | --- | --- | --- | --- | --- |
|  | start1 | stop1 | start2 | stop2 |  |  |
| 1967 | 1966-12-12 | 1967-03-05 |  |  | 83 | 0 |
| 1968 | 1967-12-08 | 1968-03-21 |  |  | 103 | 0 |
| 1969 | 1968-12-19 | 1969-04-04 |  |  | 106 | 0 |
| 1970 | 1969-12-02 | 1970-04-08 |  |  | 127 | 0 |
| 1971 | 1970-12-24 | 1971-03-19 |  |  | 85 | 0 |
| 1972 | 1972-01-10 | 1972-03-03 |  |  | 52 | 0 |
| 1973 | 1973-01-20 | 1973-02-04 | 1973-02-25 | 1973-03-11 | 29 | 21 |
| 1974 | 1973-12-01 | 1974-02-06 |  |  | 36 | 0 |
| 1975 | 1974-12-16 | 1974-12-20 | 1975-02-15 | 1975-02-19 | 10 | 56 |
| 1976 | 1976-01-04 | 1976-03-27 |  |  | 82 | 0 |
| 1977 | 1976-12-16 | 1977-03-04 |  |  | 78 | 0 |
| 1978 | 1977-11-28 | 1978-03-16 |  |  | 108 | 0 |
| 1979 | 1978-12-17 | 1979-03-31 |  |  | 104 | 0 |
| 1980 | 1980-01-03 | 1980-04-02 |  |  | 89 | 0 |
| 1981 | 1981-11-29 | 1981-12-14 | 1981-01-16 | 1981-02-26 | 56 | 33 |
| 1982 | 1981-11-30 | 1982-03-04 |  |  | 94 | 0 |
| 1983 | 1983-02-04 | 1983-03-06 |  |  | 31 | 0 |
| 1984 | 1983-12-11 | 1983-12-19 | 1984-01-15 | 1984-03-13 | 65 | 27 |
| 1985 | 1984-12-28 | 1985-03-22 |  |  | 84 | 0 |
| 1986 | 1985-12-17 | 1986-03-25 |  |  | 98 | 0 |
| 1987 | 1986-12-20 | 1987-03-30 |  |  | 101 | 0 |
| 1988 | 1987-12-07 | 1987-12-19 | 1988-01-30 | 1988-03-30 | 71 | 42 |
| 1989 | 1988-11-29 | 1988-12-23 |  |  | 24 | 0 |
| 1990 | 1989-11-26 | 1989-12-17 |  |  | 21 | 0 |
| 1991 | 1991-02-04 | 1991-03-13 |  |  | 38 | 0 |
| 1992 | 1991-12-06 | 1992-01-02 | 1992-02-04 | 1992-02-24 | 47 | 33 |
| 1993 | 1993-01-27 | 1993-03-16 |  |  | 48 | 0 |
| 1994 | 1994-02-08 | 1994-03-10 |  |  | 30 | 0 |
| 1995 | 1995-01-11 | 1995-01-23 |  |  | 12 | 0 |
| 1996 | 1995-12-11 | 1996-04-18 |  |  | 128 | 0 |
| 1997 | 1996-12-22 | 1997-01-15 |  |  | 24 | 0 |
| 1998 | 1997-11-20 | 1998-12-09 | 1998-01-31 | 1998-02-09 | 28 | 53 |
| 1999 | 1998-12-05 | 1998-12-20 | 1999-01-10 | 1999-03-01 | 65 | 21 |
| 2000 | 1999-11-19 | 2000-01-05 | 2000-01-19 | 2000-02-03 | 62 | 14 |
| 2001 | 2000-12-26 | 2001-01-08 | 2001-02-22 | 2001-03-06 | 27 | 43 |
| 2002 | 30.11.2001 | 26.01.2003 |  |  | 57 | 0 |
| 2003 | 03.12.2002 | 15.01.2003 | 01.02.2003 | 11.03.2003 | 81 | 17 |
| 2004 | 01.01.2004 | 16.03.2004 |  |  | 74 | 0 |
| 2005 | 24.11.2004 | 02.12.2004 | 21.01.2005 | 24.03.2005 | 70 | 50 |
| 2006 | 17.12.2005 | 27.03.2006 |  |  | 100 | 0 |
| 2007 | 25.01.2007 | 03.03.2007 |  |  | 37 | 0 |
| 2008 | 07.01.2008 | 19.01.2008 |  |  | 12 | 0 |
| 2009 | 04.01.2009 | 24.01.2009 | 12.02.2009 | 27.03.2009 | 63 | 19 |
| 2010 | 18.12.2009 | 19.03.2010 |  |  | 91 | 0 |
| 2011 | 30.11.2010 | 11.02.2011 |  |  | 73 | 0 |
| 2012 | 15.01.2012 | 25.02.2012 |  |  | 41 | 0 |
| 2013 | 04.12.2012 | 28.12.2012 | 11.01.2013 | 12.04.2013 | 115 | 14 |
| 2014 | 07.12.2013 | 12.12.2013 | 15.01.2014 | 08.02.2014 | 29 | 34 |
| 2015 | 21.11.2014 | 12.12.2014 |  |  | 21 | 0 |
| 2016 | 14.01.2016 | 27.01.2016 |  |  | 13 | 0 |
| 2017 | 04.01.2017 | 21.02.2017 |  |  | 48 | 0 |

Table S2. Mean monthly ambient temperatures in November-December (autumn moult of weasels), mean ambient temperatures March-April (spring moult of weasels) in the Białowieża Forest in years 1967-2017.

| Year | November | December | Mean November-December | March | April | Mean March-April |
| --- | --- | --- | --- | --- | --- | --- |
| 1967 | 3.70 | -3.00 | 0.35 | 2.75 | 7.11 | 4.93 |
| 1968 | 1.70 | -4.94 | -1.62 | 1.10 | 8.12 | 4.61 |
| 1969 | 4.47 | -9.14 | -2.33 | -4.16 | 5.34 | 0.59 |
| 1970 | 3.06 | -0.92 | 1.07 | -0.65 | 6.01 | 2.68 |
| 1971 | 1.12 | 2.02 | 1.57 | -1.37 | 7.12 | 2.88 |
| 1972 | 3.09 | -0.84 | 1.13 | 1.92 | 8.21 | 5.07 |
| 1973 | 0.55 | -2.35 | -0.90 | 2.60 | 7.27 | 4.94 |
| 1974 | 2.45 | 1.08 | 1.77 | 2.82 | 6.64 | 4.73 |
| 1975 | 0.49 | -0.29 | 0.10 | 3.86 | 7.19 | 5.53 |
| 1976 | 3.53 | -1.83 | 0.85 | -2.32 | 7.74 | 2.71 |
| 1977 | 3.72 | -2.46 | 0.63 | 3.33 | 6.34 | 4.84 |
| 1978 | 4.92 | -7.20 | -1.14 | 2.24 | 6.49 | 4.36 |
| 1979 | 1.73 | -0.08 | 0.83 | 0.57 | 6.48 | 3.53 |
| 1980 | 0.67 | -1.52 | -0.42 | -3.40 | 6.36 | 1.48 |
| 1981 | 2.14 | -3.66 | -0.76 | 2.07 | 5.16 | 3.62 |
| 1982 | 4.05 | 0.48 | 2.26 | 2.33 | 5.95 | 4.14 |
| 1983 | 1.31 | -1.63 | -0.16 | 2.59 | 9.91 | 6.25 |
| 1984 | 1.04 | -2.06 | -0.51 | 0.14 | 9.51 | 4.82 |
| 1985 | -0.19 | -0.90 | -0.55 | -0.10 | 8.01 | 3.95 |
| 1986 | 4.07 | -1.79 | 1.14 | 0.91 | 9.21 | 5.06 |
| 1987 | 2.75 | -0.81 | 0.97 | -4.00 | 6.33 | 1.16 |
| 1988 | -1.13 | -1.52 | -1.33 | -0.10 | 7.11 | 3.51 |
| 1989 | 0.50 | -0.19 | 0.15 | 4.59 | 9.67 | 7.13 |
| 1990 | 3.84 | -0.79 | 1.53 | 5.97 | 9.03 | 7.50 |
| 1991 | 3.73 | -2.31 | 0.71 | 2.81 | 7.78 | 5.30 |
| 1992 | 2.29 | -0.78 | 0.75 | 2.97 | 6.43 | 4.70 |
| 1993 | -3.86 | 0.95 | -1.46 | -0.04 | 8.49 | 4.22 |
| 1994 | 1.82 | -0.89 | 0.47 | 1.99 | 9.12 | 5.56 |
| 1995 | -0.92 | -6.50 | -3.71 | 2.32 | 8.22 | 5.27 |
| 1996 | 5.46 | -6.60 | -0.57 | -2.35 | 7.87 | 2.76 |
| 1997 | 2.45 | -2.54 | -0.04 | 2.00 | 5.14 | 3.57 |
| 1998 | -3.77 | -2.65 | -3.21 | 1.16 | 9.73 | 5.45 |
| 1999 | 0.16 | -0.25 | -0.04 | 3.64 | 9.77 | 6.70 |
| 2000 | 5.46 | 0.65 | 3.05 | 2.48 | 12.20 | 7.34 |
| 2001 | 1.56 | -6.64 | -2.54 | 1.80 | 8.88 | 5.34 |
| 2002 | 2.80 | -8.39 | -2.80 | 4.07 | 8.76 | 6.42 |
| 2003 | 4.44 | 0.23 | 2.33 | 1.31 | 6.95 | 4.13 |
| 2004 | 2.31 | 1.14 | 1.72 | 2.89 | 8.43 | 5.66 |
| 2005 | 2.15 | -1.59 | 0.28 | -1.39 | 8.73 | 3.67 |
| 2006 | 4.51 | 3.13 | 3.82 | -1.68 | 8.62 | 3.47 |
| 2007 | 0.74 | -0.56 | 0.09 | 6.20 | 8.73 | 7.47 |
| 2008 | 3.70 | 0.27 | 1.99 | 2.50 | 9.53 | 6.02 |
| 2009 | 3.78 | -2.85 | 0.46 | 2.48 | 13.77 | 8.13 |
| 2010 | 4.68 | -6.26 | -0.79 | 3.39 | 11.50 | 7.44 |
| 2011 | 2.80 | 1.53 | 2.17 | 3.13 | 11.33 | 7.23 |
| 2012 | 5.05 | -4.84 | 0.11 | 4.61 | 10.10 | 7.36 |
| 2013 | 4.72 | 1.05 | 2.88 | -2.29 | 8.43 | 3.07 |
| 2014 | 2.13 | -0.87 | 0.63 | 7.55 | 11.20 | 9.37 |
| 2015 | 4.12 | 3.47 | 3.79 | 6.45 | 9.40 | 7.93 |
| 2016 | 1.85 | -0.29 | 0.78 | 4.76 | 9.80 | 7.28 |
| 2017 | 3.90 | 1.48 | 2.69 | 5.87 | 7.60 | 6.74 |
