# Supplementary material for: Climate change is affecting mortality of weasels due to camouflage mismatch
Source: Sci Rep. 2018 May 24;8:7648. doi: 10.1038/s41598-018-26057-5 (PMC5967304; doi:10.1038/s41598-018-26057-5)
Supplement: Supplementary file 2 — Supplementary information [file 41598_2018_26057_MOESM2_ESM.pdf]

Supplementary information

Climate change is affecting mortality of weasels due to camouflage mismatch

Kamal Atmeh, Anna Andruszkiewicz, Karol Zub

Fig. S1. Weasels *Mustela nivalis nivalis* and *Mustela nivalis vulgaris* in summer in winter coat, matching and mismatching background colour. All pictures by Karol Zub.

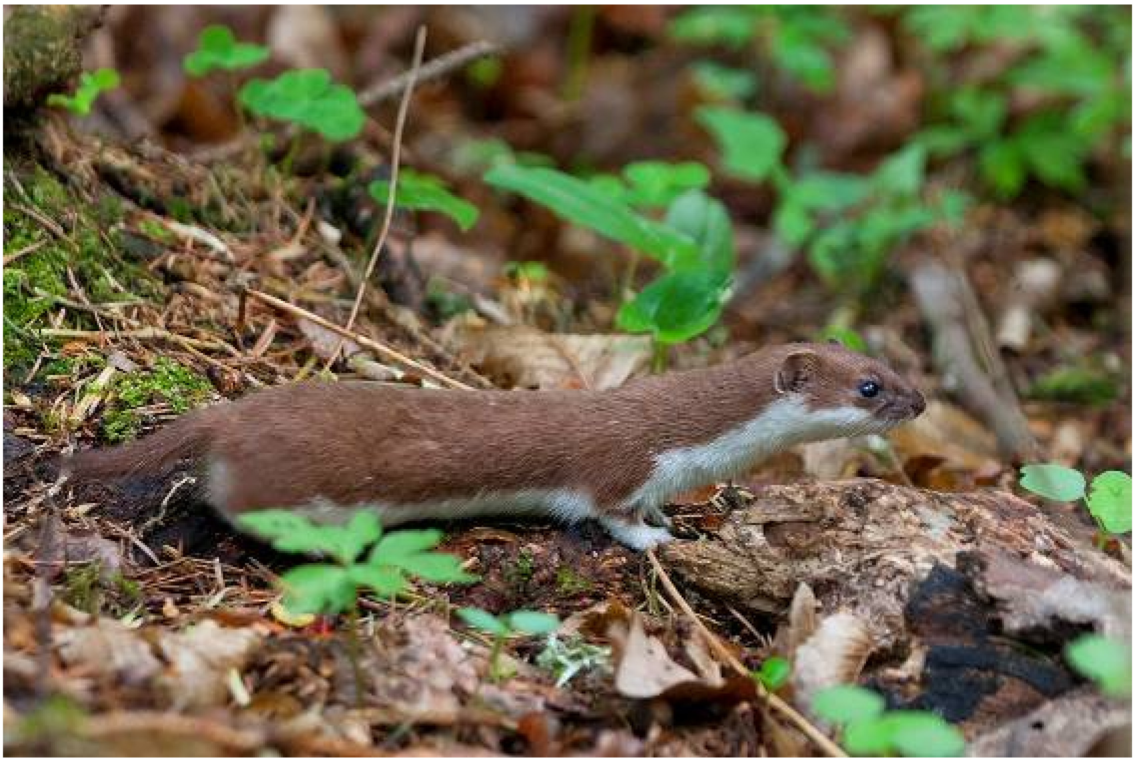

A. *Mustela nivalis nivalis* in full summer coat

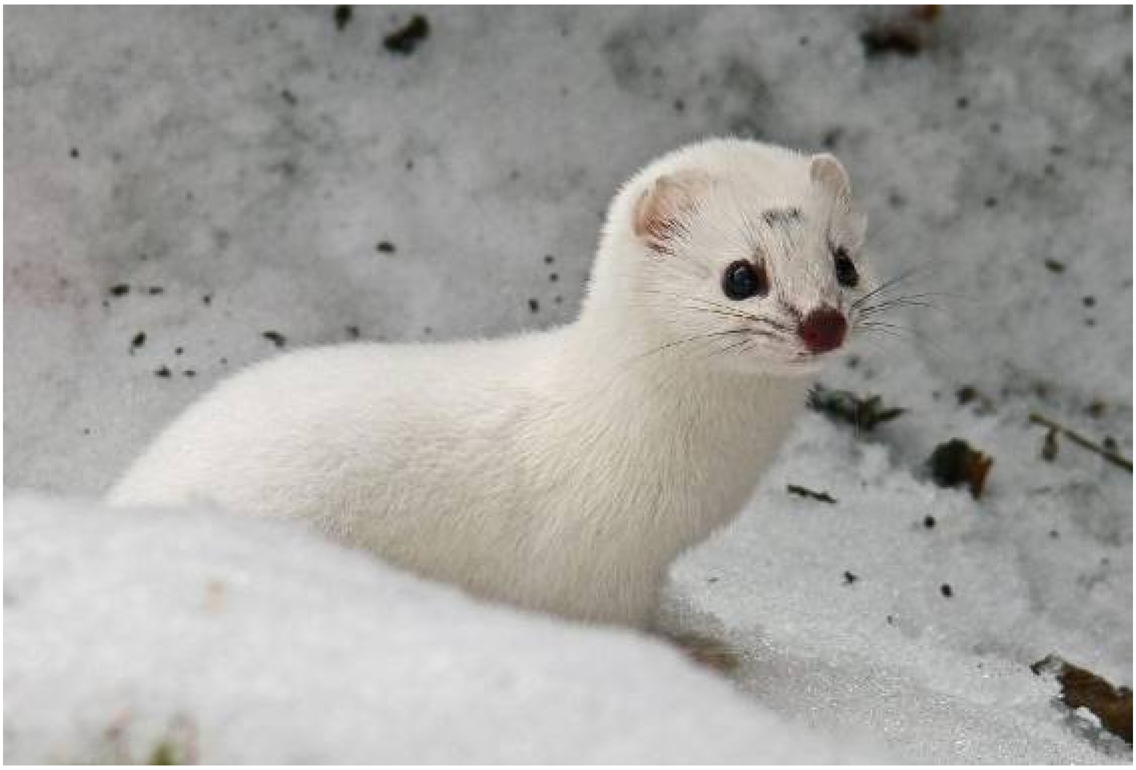

B. *Mustela nivalis nivalis* in full winter coat

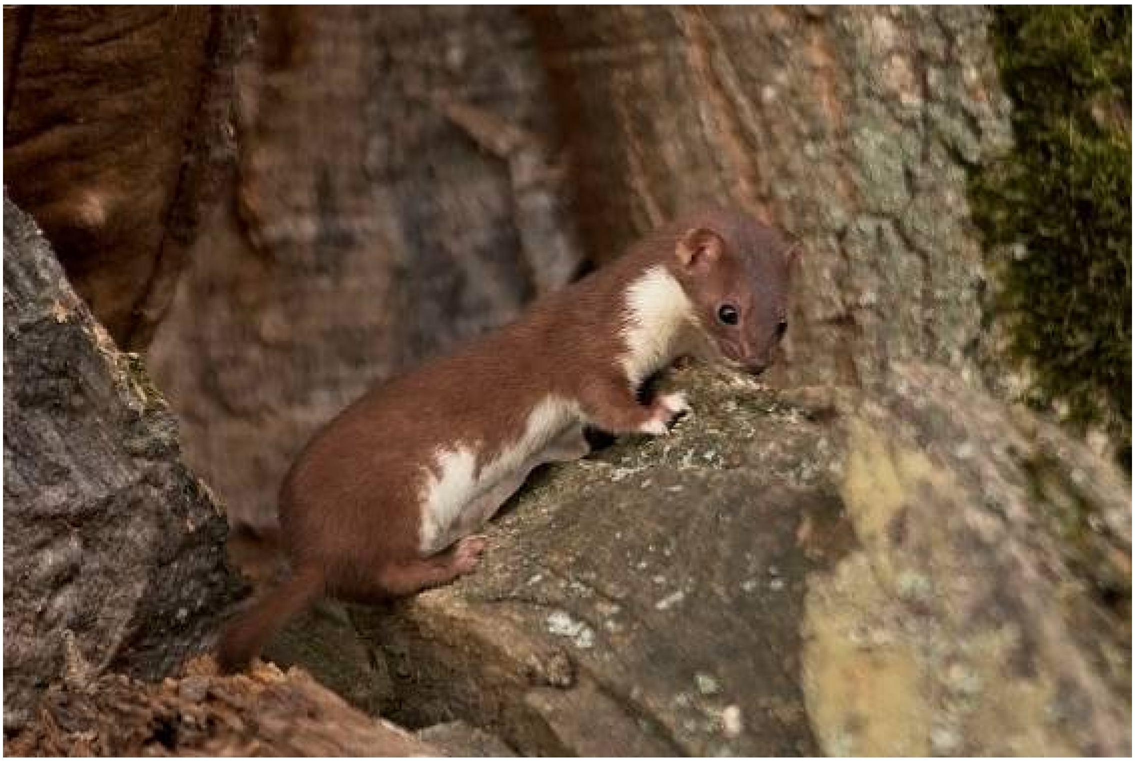

C. *Mustela nivalis vulgaris* in full summer coat

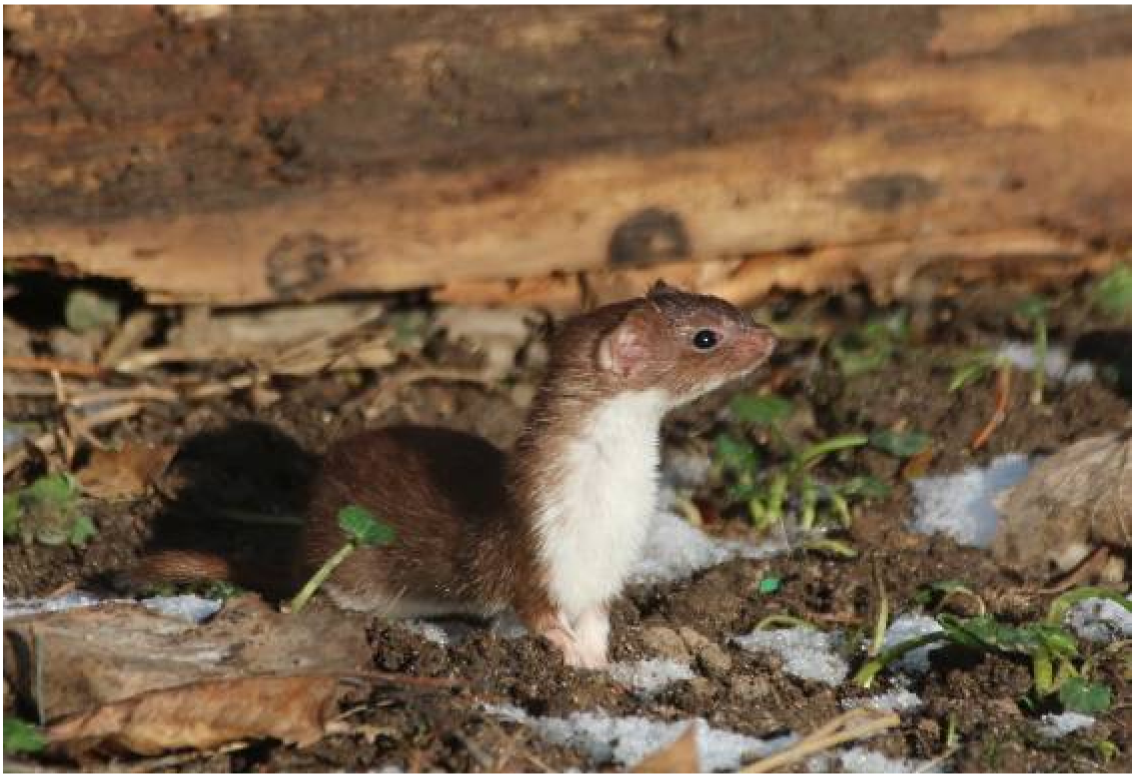

D. *Mustela nivalis vulgaris* in full winter coat

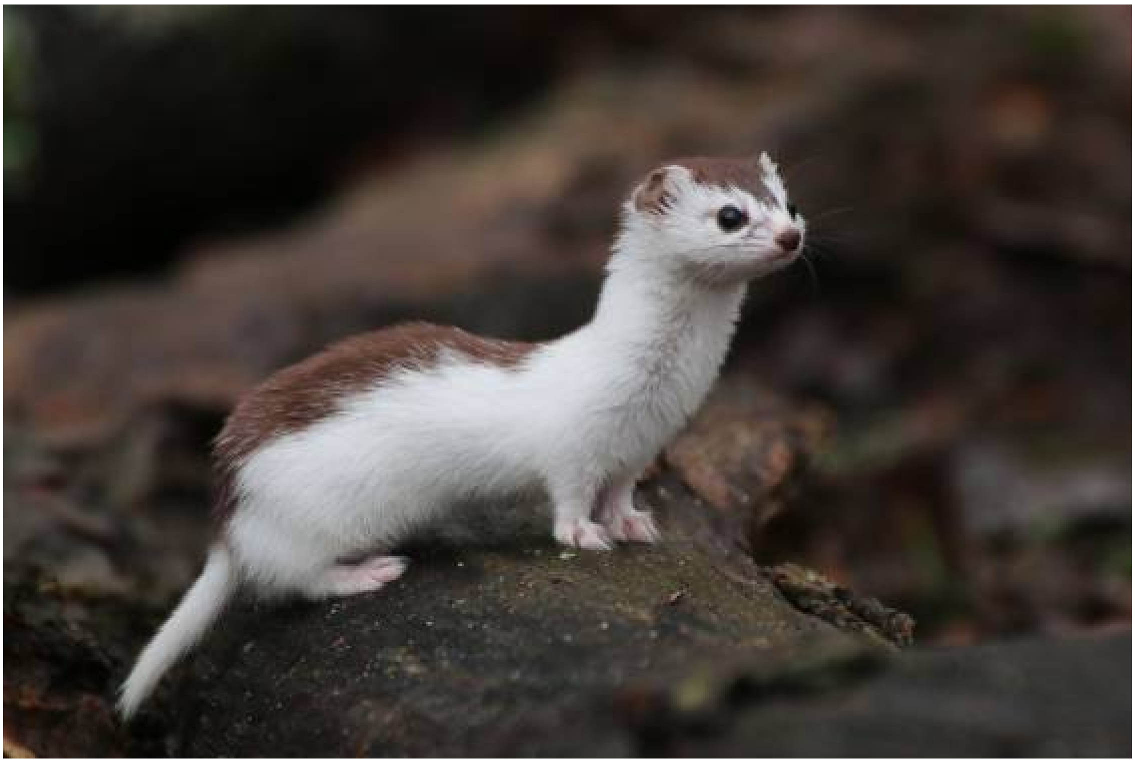

E. *Mustela nivalis nivalis* in winter coat (last phase of moult)

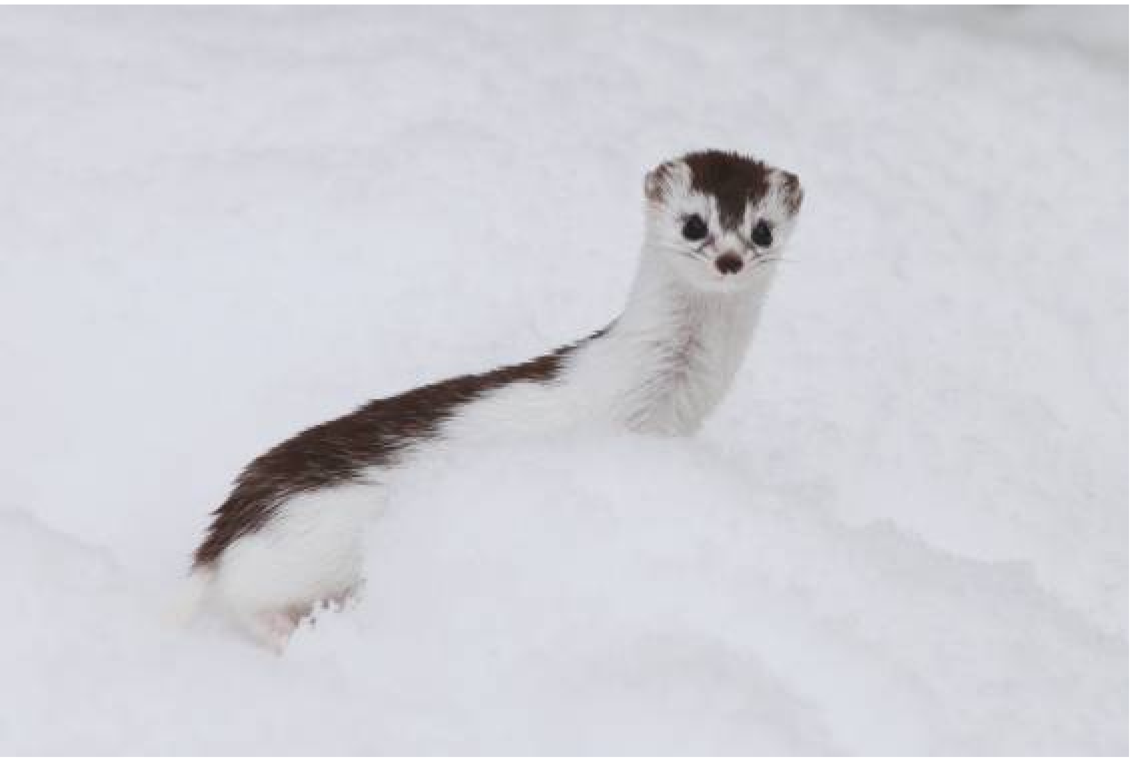

F. *Mustela nivalis nivalis* in winter coat (last phase of moult)

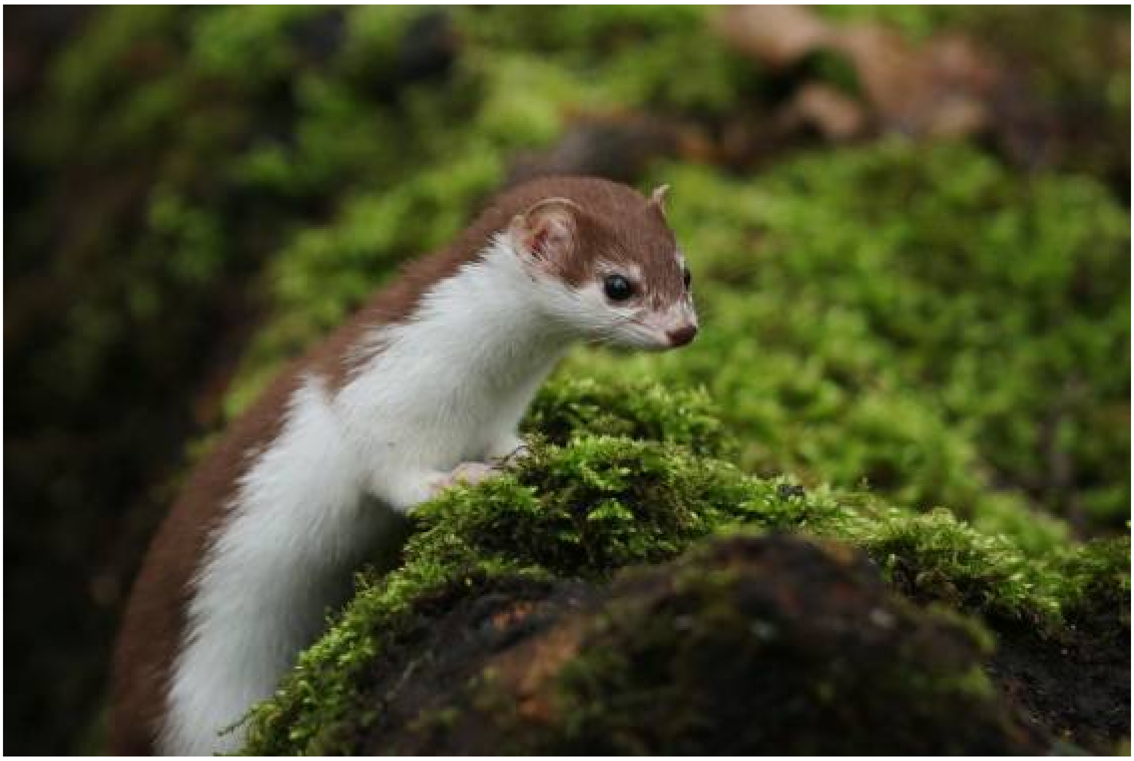

G. *Mustela nivalis nivalis* in winter coat (partly moulted)

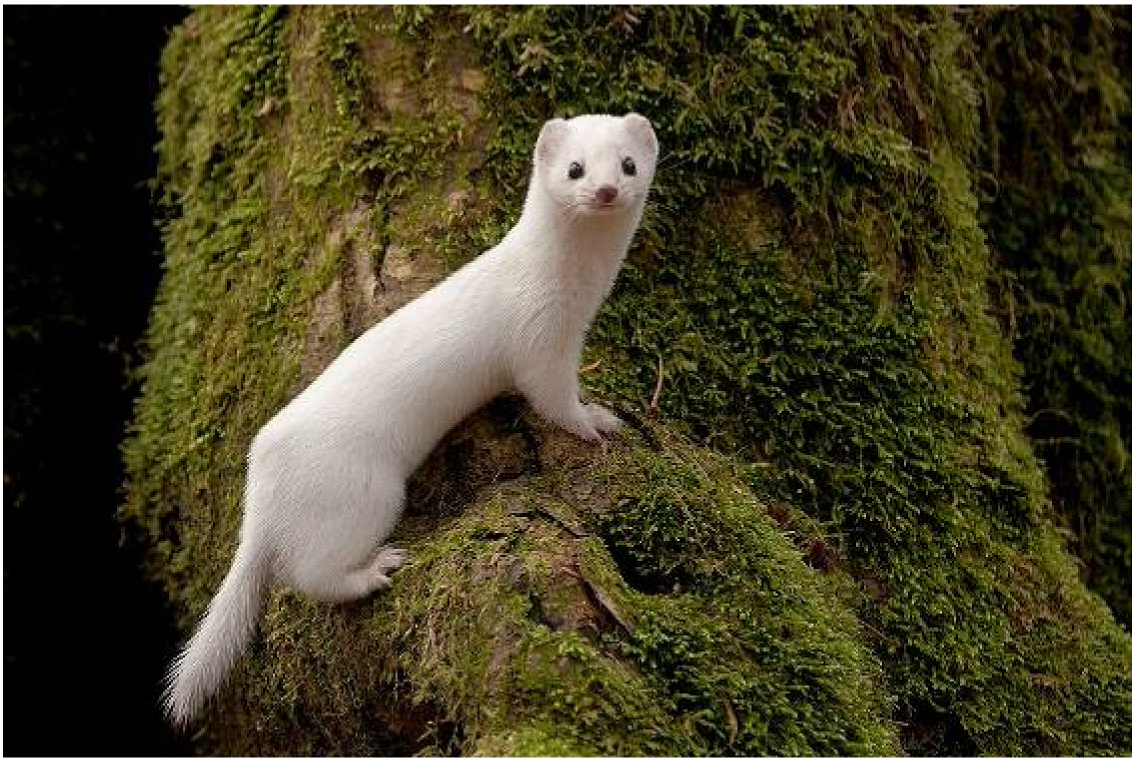

H. *Mustela nivalis nivalis* in full winter coat

Fig. S2. Moult phases of *Mustela nivalis nivalis*. Pictures show live, sedated individuals. All pictures by Karol Zub.

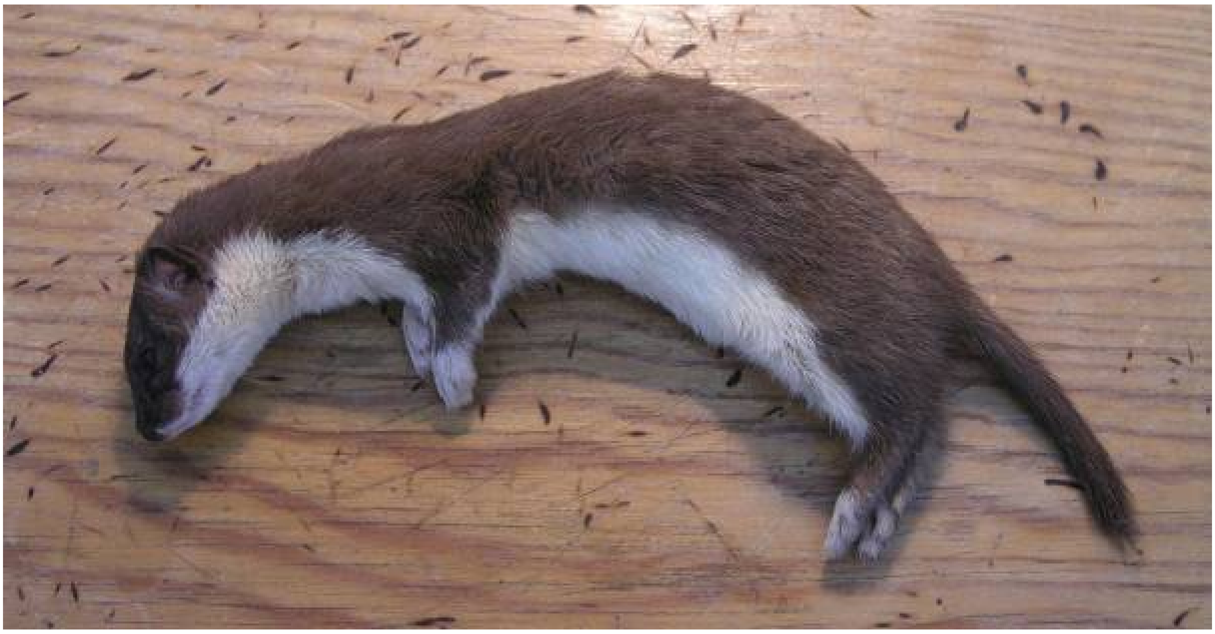

A. Summer coat (dorsal part of body 100% brown)

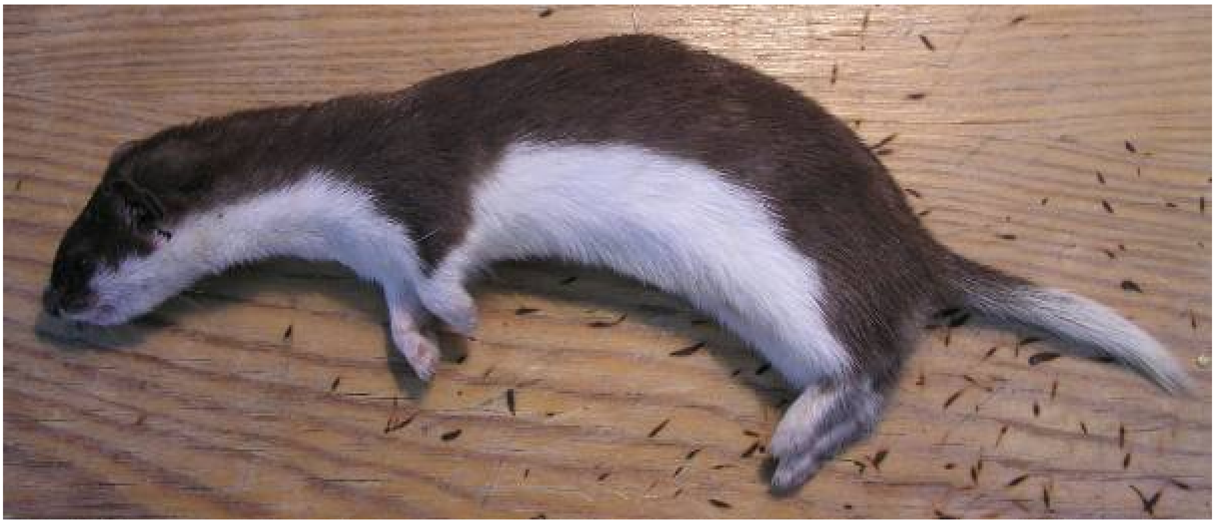

B. Beginning of moult (dorsal part of body > 75% brown)

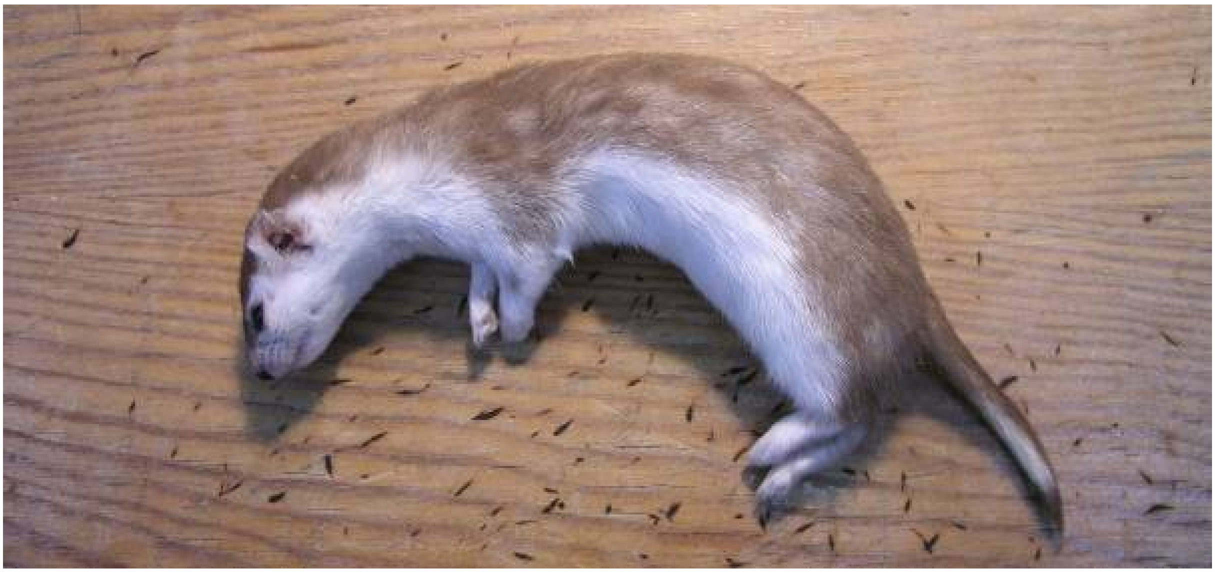

C1. Advanced moult (dorsal part of body 25-75% brown)

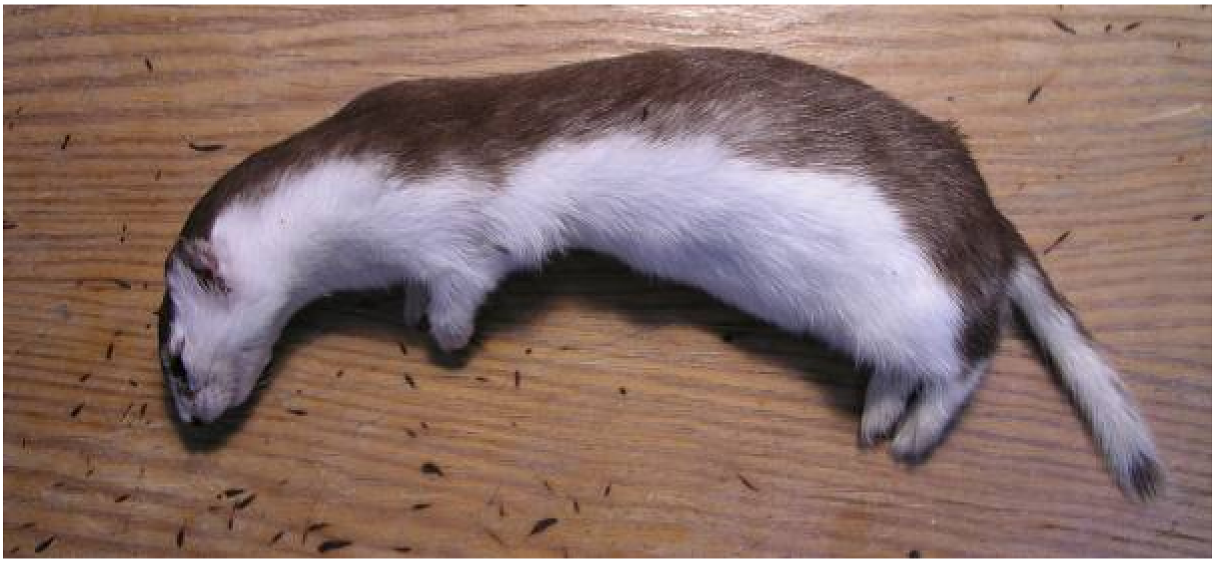

C2. Advanced moult (dorsal part of body 25-75% brown)

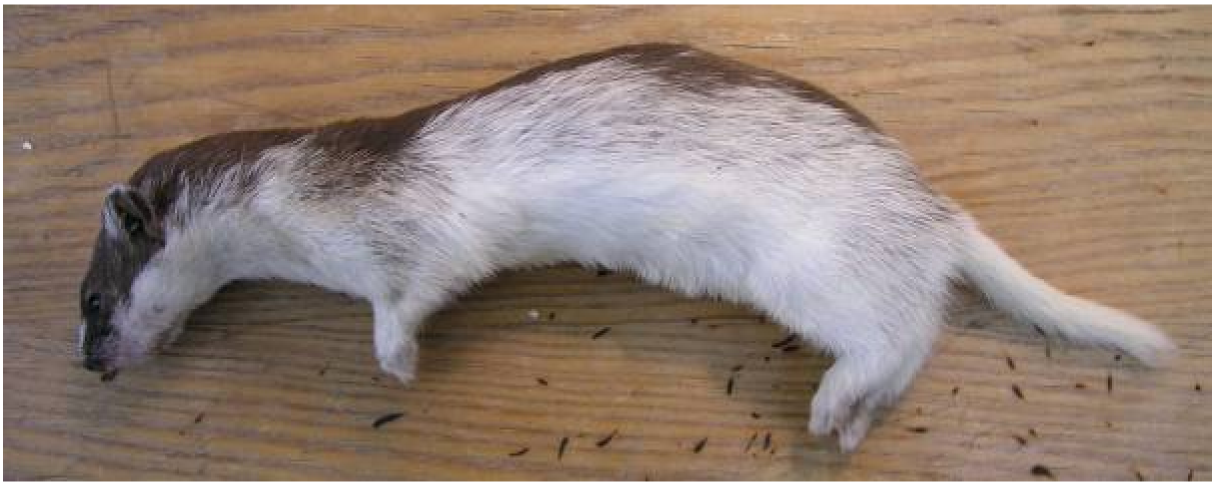

D1. Last phase of moult (dorsal part of body < 25% brown)

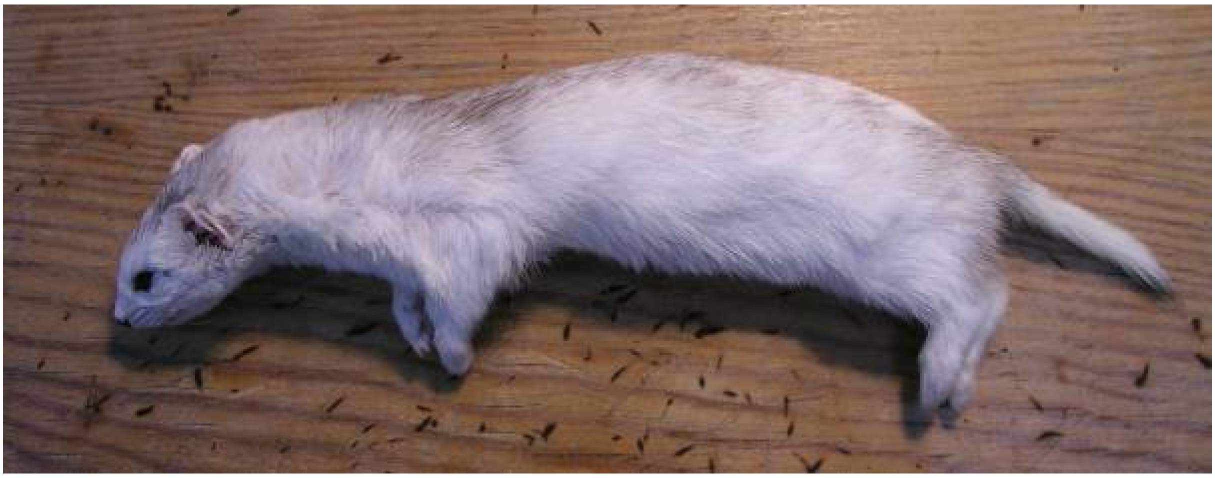

D2. Last phase of moult (dorsal part of body < 25% brown)

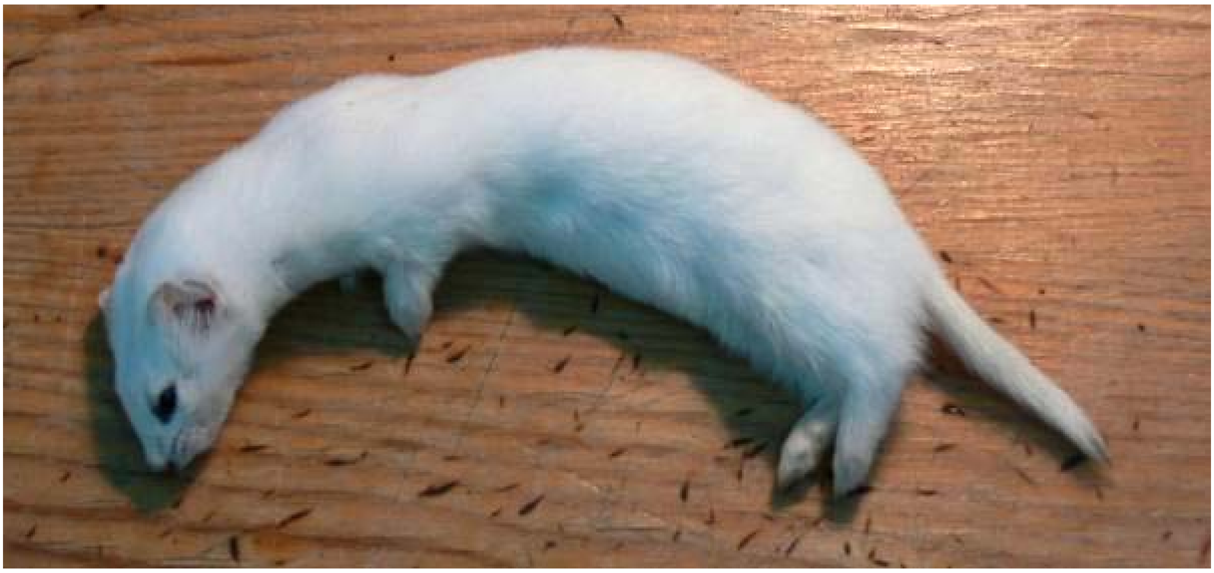

E. Winter coat (dorsal part of body 100% white)

Fig. S3. Experimental design

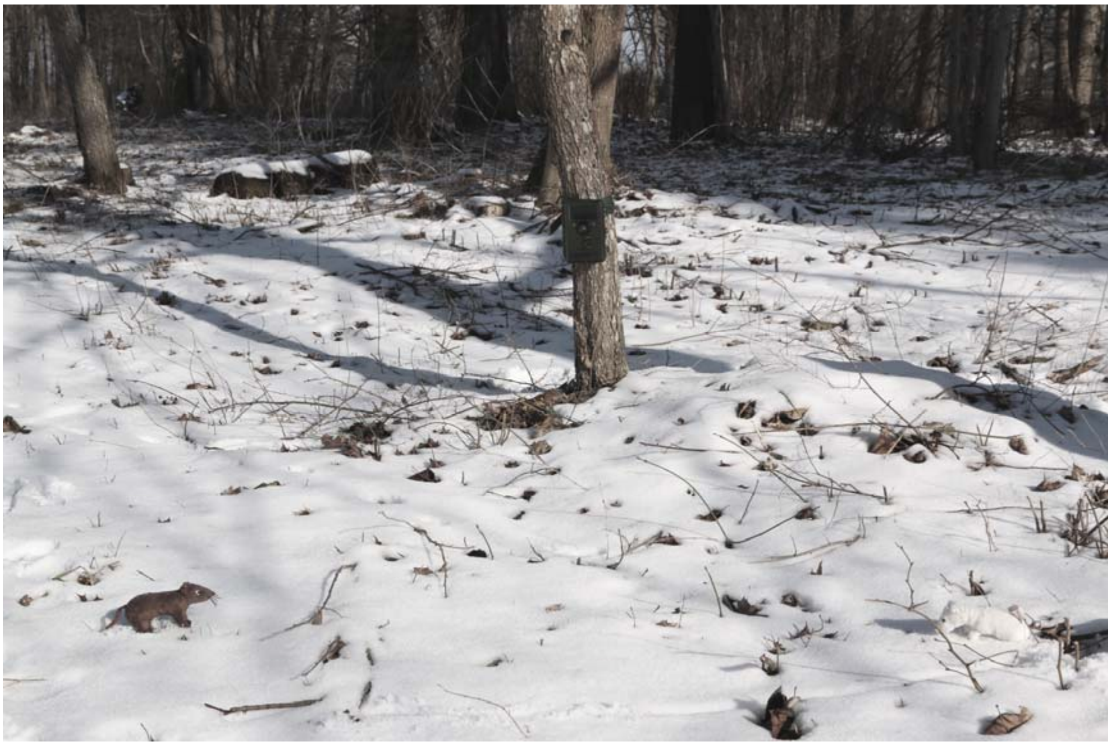

A. Experimental design – models exposed on the snow and camera-trap.

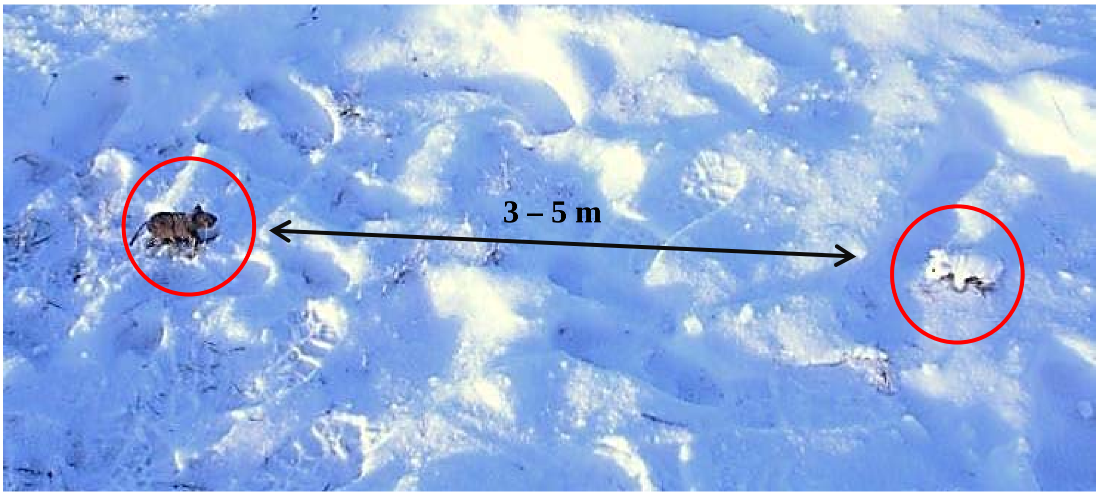

B. Details of experimental design - brown model (left) and white model (right) on the snow during the experiment.

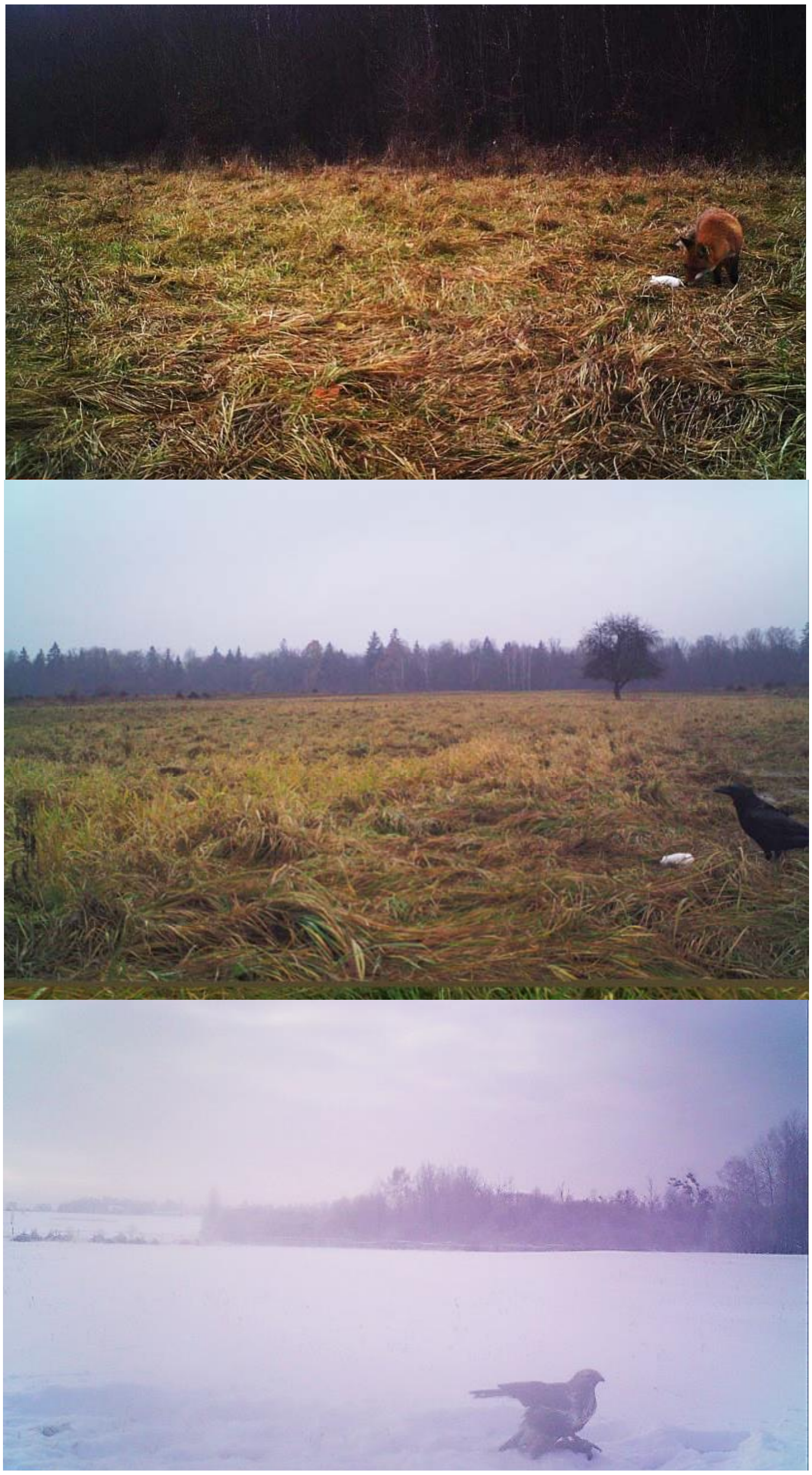

C. Photographs from camera-traps presenting model detection by predators - red fox sniffing white model (upper panel), raven observing white model (middle panel), and rough-legged buzzard after attack on brown model (lower panel).
